# Comprehensive Evaluation of One-Carbon Metabolism Pathway Gene Variants and Renal Cell Cancer Risk

**DOI:** 10.1371/journal.pone.0026165

**Published:** 2011-10-19

**Authors:** Todd M. Gibson, Paul Brennan, Summer Han, Sara Karami, David Zaridze, Vladimir Janout, Helen Kollarova, Vladimir Bencko, Marie Navratilova, Neonila Szeszenia-Dabrowska, Dana Mates, Alena Slamova, Ruth M. Pfeiffer, Rachael Z. Stolzenberg-Solomon, Susan T. Mayne, Meredith Yeager, Stephen Chanock, Nat Rothman, Wong-Ho Chow, Philip S. Rosenberg, Paolo Boffetta, Lee E. Moore

**Affiliations:** 1 Division of Cancer Epidemiology and Genetics, National Cancer Institute, Bethesda, Maryland, United States of America; 2 Yale School of Public Health, New Haven, Connecticut, United States of America; 3 International Agency for Research on Cancer, Lyon, France; 4 Institute of Carcinogenesis, Cancer Research Centre, Moscow, Russia; 5 Department of Preventive Medicine, Faculty of Medicine, Palacky University, Olomouc, Czech Republic; 6 1st Faculty of Medicine, Institute of Hygiene and Epidemiology, Charles University, Prague, Czech Republic; 7 Department of Cancer Epidemiology and Genetics, Masaryk Memorial Cancer Institute, Brno, Czech Republic; 8 Department of Epidemiology, Institute of Occupational Medicine, Lodz, Poland; 9 Institute of Public Health, Bucharest, Romania; 10 Core Genotyping Facility, National Cancer Institute, Gaithersburg, Maryland, United States of America; 11 The Tisch Cancer Institute, Mount Sinai School of Medicine, New York, New York, United States of America; 12 International Cancer Prevention Research Institute, Lyon, France; Instituto de Investigación Sanitaria INCLIVA, Spain

## Abstract

**Introduction:**

Folate and one-carbon metabolism are linked to cancer risk through their integral role in DNA synthesis and methylation. Variation in one-carbon metabolism genes, particularly *MTHFR*, has been associated with risk of a number of cancers in epidemiologic studies, but little is known regarding renal cancer.

**Methods:**

Tag single nucleotide polymorphisms (SNPs) selected to produce high genomic coverage of 13 gene regions of one-carbon metabolism (*ALDH1L1*, *BHMT*, *CBS*, *FOLR1*, *MTHFR*, *MTR*, *MTRR*, *SHMT1*, *SLC19A1*, *TYMS*) and the closely associated glutathione synthesis pathway (*CTH*, *GGH*, *GSS*) were genotyped for 777 renal cell carcinoma (RCC) cases and 1,035 controls in the Central and Eastern European Renal Cancer case-control study. Associations of individual SNPs (n = 163) with RCC risk were calculated using unconditional logistic regression adjusted for age, sex and study center. Minimum p-value permutation (Min-P) tests were used to identify gene regions associated with risk, and haplotypes were evaluated within these genes.

**Results:**

The strongest associations with RCC risk were observed for *SLC19A1* (P_min-P_ = 0.03) and *MTHFR* (P_min-P_ = 0.13). A haplotype consisting of four SNPs in *SLC19A1* (rs12483553, rs2838950, rs2838951, and rs17004785) was associated with a 37% increased risk (p = 0.02), and exploratory stratified analysis suggested the association was only significant among those in the lowest tertile of vegetable intake.

**Conclusions:**

To our knowledge, this is the first study to comprehensively examine variation in one-carbon metabolism genes in relation to RCC risk. We identified a novel association with *SLC19A1*, which is important for transport of folate into cells. Replication in other populations is required to confirm these findings.

## Introduction

One-carbon metabolism refers to a system of interdependent metabolic pathways that facilitate the transfer of one-carbon units and ultimately provide the precursors needed for DNA synthesis and repair as well as DNA methylation. Interconversion of various forms of folate is at the foundation of one-carbon metabolism, and folate deficiency has been shown to cause DNA strand breaks, diminished DNA repair capacity, and aberrant methylation patterns [1]. Folate intake has been associated with risk of a number of cancers, including those of the colorectum, esophagus, stomach, and pancreas [2]–[4]. Little is known about the associations between one-carbon metabolism and renal cancer, though one study suggested folate deficiency is associated with increased risk [5].

A candidate gene analysis by Moore et al. in a large renal cell carcinoma (RCC) case-control study conducted in Central and Eastern Europe examined 10 SNPs in 5 one-carbon metabolism genes and found an association between the *MTHFR* 677C>T polymorphism and RCC risk [6]. This polymorphism, which results in reduced enzyme activity, has previously been associated with various cancers, with the most consistent evidence pointing to a reduced risk of colorectal cancer [7]–[10]. Other genes involved in one-carbon metabolism reactions include *TYMS*, *MTR*, *MTRR*, *BHMT*, *SHMT*, and *ALDH1L1* (Table 1, Figure S1). The *FOLR1*, *SLC19A1* and *GGH* genes may also be important since they are involved in folate transport and availability. *CBS*, *CTH* and *GSS* are part of a related pathway in which homocysteine is converted to glutathione, which has been shown to modify cancer risk due to its role in antioxidant reactions and Phase II xenobiotic metabolism [11]. The association of these genes with renal cancer risk is unknown.

**Table 1:** Individual SNP and gene-based global minimum p-values for candidate one-carbon metabolism genes and risk of renal cell carcinoma.

Gene Name (*alias*)	Gene Function	Chromosome Location	Number of Tag SNPs	Minimum P-trend^1^^,^ 2	FDR-adjusted P-trend^1^^,^ 3	Adjusted Min-P Test
[**1**] ** SLC19A1**: Solute Carrier Family 19 (folate transporter), member 1 (*CHMD*, *FOLT*, *IFC1*, *REFC*, *RFC1*)	Carrier-mediated transport of folate compounds into cells; plays a role in maintaining intracellular concentrations of folate.	21q22.3	6	0.01	0.03	0.032
[**2**] ** MTHFR**: 5,10-Methylenetetrahydrofolate Reductase (NADPH)	Irreversible conversion of 5,10-methylenetetrahydrofolate to 5-methylenetetrahydrofolate, which then participates in conversion of homocysteine to methionine and subsequently S-adenosylmethionine; key regulatory enzyme in folate metabolism.	1p36.3	8	0.02	0.16	0.130
[**3**] ** BHMT**: Betaine-Homocysteine Methyltransferase	Involved in the regulation of homocysteine metabolism. Converts betaine and homocysteine to dimethylglycine and methionine, respectively.	5q13.1–q15	13	0.03	0.35	0.337
[**4**] ** MTR**: 5-Methyltetrahydrofolate-Homocysteine Methyltransferase (*MS*, *FLJ33168*, *FLJ43216*, *FLJ45386*)	Catalyzes the final step in methionine biosynthesis in a reaction that involves cobalamin (B12) and 5-methyltetrahydrofolate.	1q43	9	0.05	0.21	0.371
**[NS] GGH**: Gamma-Glutamyl Hydrolase (*conjugase, folylpolygammaglutamyl hydrolase, GH*)	Hydrolyzes the polyglutamate sidechains of pteroylpolyglutamates, the predominant form of the folate in food; may play an important role in the bioavailability of dietary pteroylpolyglutamates and in the metabolism of pteroylpolyglutamates and antifolates	8q12.3	6	0.09	0.33	0.467

Gene Name (*alias*)	Gene Function	Chromosome Location	Number of Tag SNPs	Minimum P-trend^1^^,^ 2	FDR-adjusted P-trend^1^^,^ 3	Adjusted Min-P Test
[**5**] ** TYMS**: Thymidylate Synthetase (*TS, TMS, TSase, HsT422, MGC88736*)	Catalyzes the methylation of deoxyuridylate to deoxythymidylate using 5,10-methylenetetrahydrofolate as a cofactor; maintains the dTMP pool critical for DNA replication and repair.	18p11.32	14	0.09	0.46	0.601
[**6**] ** CTH**: Cystathionase (*Cystathionine gamma-lyase, gamma cystathionase, MGC 9471*)	Converts cystathione derived from methionine into cysteine. Glutathione synthesis in the liver is dependent upon the availability of cysteine.	1p31.1	13	0.08	0.72	0.667
[**7**] ** ALDH1L1**: Aldehyde Dehydrogenase 1 Family, member L1 (*FTHFD, DKFZp781N0997*)	Catalyzes the conversion of 10-formyltetrahydrofolate, NADP, and water to tetrahydrofolate, NADPH, and carbon dioxide.	3q21.2	34	0.04	0.84	0.778
[**8**] ** FOLR1**: Folate Receptor 1 (*FBP, FOLR, MOv18, FR-alpha*)	Binds to folate and reduced folic acid derivatives and mediates delivery of 5-methyltetrahydrofolate to the interior of cells.	11q13.3–q14.1	3	0.43	0.75	0.786
[**9**] ** CBS**: Cystathionine-Beta-Synthase (*HIP-4*)	Catalyzes the conversion of homocysteine to cystathionine, the first step in the transsulfuration pathway.	21q22.3	25	0.09	0.97	0.922
[**10**] ** SHMT1**: Serine Hydroxymethyltransferase 1 (*SHMT, CSHMT, MGC 15229, MGC 24556*)	Catalyzes the reversible conversion of serine and tetrahydrofolate to glycine and 5,10-methylene tetrahydrofolate. This reaction provides one carbon units for synthesis of methionine, thymidylate, and purines.	17p11.2	5	0.51	0.89	0.970

Gene Name (*alias*)	Gene Function	Chromosome Location	Number of Tag SNPs	Minimum P-trend^1^^,^ 2	FDR-adjusted P-trend^1^^,^ 3	Adjusted Min-P Test
[**11**] ** MTRR**: 5-Methyltetrahydrofolate-Homocysteine Methyltransferase Reductase (*MSR, MGC129643*)	Involved in the reductive regeneration of cobalamin cofactor required for the maintenance of methionine synthase in a functional state	5p15.3–p15.2	16	0.27	1.00	0.988
[**12**] ** GSS**: Glutathione Synthetase (*GSHS, MGC 14098*)	Catalyzes the second step of glutathione biosynthesis; glutathione is important for a variety of biological functions, including protection of cells from oxidative damage by free radicals, detoxification of xenobiotics, and membrane transport	20q11.2	9	0.47	0.99	0.990

1 Adjusted for age, sex and study center.

2 Minimum P-value for all tagging SNPs in each gene region using an additive model.

3 FDR-adjusted minimum P-value for all tagging SNPs in each gene region using an additive model.

Note: Numbers in brackets [#] correspond to locations on Figure S1.

We have expanded on the previous findings of Moore et al. to more comprehensively investigate genetic variation across *MTHFR* and 12 additional candidate genes involved in folate transport, one-carbon metabolism and glutathione synthesis. We examined the relationship between RCC risk and 163 tag SNPs that provided high genomic coverage of 13 target gene regions among RCC cases and controls from Central Europe, an area with some of the highest rates of RCC incidence and mortality worldwide [12].

## Materials and Methods

### Study Population

The Central and Eastern European Renal Cell Cancer (CEERCC) Study, details of which have been described previously [13], is a multi-center, hospital-based case-control study conducted from August 1999 to January 2003. Cases consisted of 1,097 patients newly diagnosed with histologically-confirmed RCC, ages 20–79, recruited from centers in Russia (Moscow), Romania (Bucharest), Poland (Lodz) and Czech Republic (Prague, Olomouc, Ceske Budejovice, Brno). Diagnostic information was extracted from hospital records by trained medical staff. Eligible controls included 1,497 patients admitted to the same hospital as cases, but for conditions not related to smoking or genitourinary disorders. Controls were frequency matched to cases based on age (±3 years), sex and study center (including hospital of recruitment), and both cases and controls were required to have been residents of the study center area for at least one year at the time of recruitment. Response rates ranged from 90.0–98.6% for cases and from 90.3–96.1% for controls, and all study participants were Caucasian. Blood samples were collected and genomic DNA, extracted from whole blood buffy coat using a standard phenol-chloroform extraction method, was obtained for 987 cases (90%) and 1,298 controls (88%). Written informed consent was obtained from all patients and their physicians, and the study was approved by the institutional review boards of the National Cancer Institute (NCI), the International Agency for Research on Cancer (IARC), and each study center.

### Data Collection

Trained personnel conducted in-person interviews and used standardized questionnaires to collect information on demographics, education, tobacco smoking, diet, anthropometric measures, and medical, family and occupation histories. All cases were interviewed within three months of RCC diagnosis. Assessment of dietary intake has been described previously [14]. Briefly, the food frequency questionnaire (FFQ) consisted of 23 food items/categories, selected with local investigators to capture consumption patterns in these specific regions. Frequency of consumption of each food item was assessed using six categories, ranging from never to daily. Food group categories were formed on the basis of intake of similar foods (e.g. cruciferous vegetables included brussel sprouts, broccoli and cabbage). The assessment of vegetable intake, designed to comprehensively cover intake in this region through 1999 [14], was based on the eight specific vegetables commonly eaten in the region (carrots, cabbage, spinach, broccoli, brussel sprouts, onion, pumpkin, tomatoes) plus an additional category for “other fresh and preserved vegetables”. Tertiles of vegetable intake were calculated based on the consumption frequencies reported among controls.

### Genotyping

Genotyping was performed at the NCI's Core Genotyping Facility (CGF) using an Illumina GoldenGate® Oligo Pool All (OPA) assay. CGF staff was blinded to case-control status and samples were blinded and randomized on PCR plates. Duplicate genotyping was performed for a randomly selected 5% of the samples for quality control. Genotyping was performed on a subset of 777 cases (70.8%) and 1,035 controls (70.1%) who provided sufficient quality and quantity of DNA required for use of the Illumina® OPA assay. The genotyping completion rate was greater than 98% for all SNPs except rs6519519 (97%). The quality control concordance rate was 98% or greater for all SNPs except rs1476413 (94%), rs11121832 (96%), rs234712 (95%), rs4646768 (97%), and rs13069815 (96%). Nine candidate SNPs (Table S1) were genotyped previously at CGF using a validated TaqMan assay, details of which can be found at http://snp500cancer.nci.nih.gov/home.cfm. Methods and results for these nine SNPs, previously reported by Moore et al. [5], were included in our analyses for gene-based tests as well as the identification and testing of haplotypes in multivariable and stratified analyses.

Genes were selected *a priori* based on their role in one-carbon metabolism or the closely associated glutathione synthesis pathway (*CBS*, *CTH* and *GSS*). Genotypes were obtained for 167 tag SNPs selected to provide high genomic coverage (80–90%) for 13 candidate genes involved in one-carbon metabolism and glutathione synthesis (see Table 1, Figure S1). Tag SNPs were selected from among common variants (minor allele frequencies ≥5%) found in Caucasians using a tag SNP method [15] with an r^2^>0.80. The regions 20 kb upstream of the transcription start site and 10 kb downstream of the last exon using HapMap CEU data (http://www.hapmap.org) were included for tag SNP selection to ensure thorough coverage of each target gene. Nonsynonymous SNPs and those with putative functional significance were also included in our analysis. All SNPs included in this study are reported in the National Cancer Institute's SNP500Cancer database (http://snp500cancer.nci.nih.gov) [16]. No deviations from expected Hardy-Weinberg proportions (chi-square tests, p<0.05 for deviation) were observed for genotype frequencies among the controls.

### Statistical Analyses

A sequence of analyses was used to comprehensively examine the associations of single SNPs, genes, gene regions, and haplotypes with RCC risk while accounting for multiple testing. Odds ratios (ORs) and 95% confidence intervals (CIs) for the association of each individual SNP with RCC risk were determined using unconditional logistic regression assuming a codominant model of inheritance for SNP genotypes. For linear trend tests the homozygous common, heterozygous and homozygous rare groups were coded as 0, 1 or 2 respectively, corresponding to the number of rare alleles. Logistic regression models were adjusted for age (continuous), sex, and country. Further adjustment for smoking, self-reported hypertension or body mass index did not appreciably change the risk estimates (<5%). Multiplicative interactions between SNPs and these RCC risk factors were evaluated by likelihood ratio tests comparing models with and without interaction terms, but we found no evidence of effect modification.

Global significance of associations between individual genes and RCC risk was assessed using the minimum p-value permutation (min-P) test [17], [18], which combines information on the set of SNP-RCC associations within a gene and accounts for the correlation between SNPs. Inferences are based on the permutation distribution of the minimum p-value among the set of SNPs in a gene. Min-P tests were adjusted for multiple comparisons among genes using Benjamini and Hochberg's False Discovery Rate (FDR) procedure [19], [20]. For genes identified by the single SNP or min-P tests, a haplowalk sliding-window approach was employed in which consecutive 3 SNP windows were examined to identify chromosome regions of interest for further analysis of haplotypes. For each 3 SNP window, the Haplostats package (version 1.3.1) in R (version 2.4.1) was used to reconstruct haplotype frequencies in cases and controls using an expectation maximization (EM) algorithm and to test for association with case-control status using generalized linear models (haplo.glm) and a Wald test. SNP windows with an FDR-adjusted p-value below 0.1 were considered to denote a region of interest.

Linkage disequilibrium (LD) between SNPs among control subjects was determined using the Haploview program [21] to calculate r^2^ values. Based on the results of the haplowalk procedure and the observed LD structure, haplotype-blocks were selected for further analysis. For these blocks, haplotypes were reconstructed and associations with case-control status (ORs and 95% CIs) were evaluated in Haplostats in R, with adjustment for age (<50, 50–<60, 60–<70 and 70+ years), sex and study center. The most common haplotype served as the reference, and haplotypes with an estimated frequency of less than 5% were combined into a separate “rare” haplotype group. Further analyses examined the haplotype associations within strata of vegetable or alcohol intake (based on tertiles of consumption among controls). A likelihood ratio test comparing the fit of models with and without interaction terms was used to evaluate heterogeneity of genotype frequencies among countries, but we did not observe any evidence of heterogeneity. Statistical analyses were conducted using SAS version 9.1 (SAS Institute, Cary, NC), except where otherwise noted.

## Results

Demographic and lifestyle characteristics of all participants and genotyped participants in the CEERCC study are shown in Table 2. The distribution of RCC risk factors in successfully genotyped participants was similar to that of all participants. The majority of participants were from the Czech Republic, and cases were more likely to be from that country than controls. Cases were more likely to be obese (BMI>30 kg/m^2^) and less likely to be current smokers, although the association with smoking was no longer present after adjustment for age, sex and study center.

**Table 2:** Demographic and lifestyle characteristics of participants in the Central and Eastern European Renal Cell Cancer Study.

	All Participants					Genotyped Participants				
	Cases		Controls			Cases		Controls		
	n	%	n	%	P-val^1^	n	%	n	%	P-val^1^
**Total**	1,097		1,476			777		1,035		
**Center**										
Bucharest, Romania	95	8.7	160	10.8		68	8.8	94	9.1	
Lodz, Poland	99	9.0	198	13.4		80	8.7	189	18.3	
Moscow, Russia	317	28.9	463	31.4		242	31.1	313	30.2	
Czech Republic^2^	586	53.4	655	44.4	<0.001	387	49.8	439	42.4	<0.001
**Sex**										
Male	648	59.1	952	64.5		472	60.8	648	62.6	
Female	449	40.9	524	35.5	0.01	305	39.3	387	37.4	0.42
**Mean age ± Std Dev (yrs)**	59.6±10.3		59.3±10.3		0.61	59.5±10.4		59.0±10.2		0.34
**Body Mass Index (kg/m2)**										
<25	327	29.8	532	36.2		222	28.6	375	36.3	
25–30	476	43.4	620	42.2		330	42.5	432	41.9	
>30	293	26.7	318	21.6	0.001	225	29.0	225	21.8	<0.001
**Smoking Status**										
Never	510	46.6	599	40.7		359	46.4	420	40.7	
Former	251	22.9	353	24.0		174	22.5	246	23.8	
Current	333	30.4	521	35.4	0.01	241	31.1	367	35.5	0.04
**Hypertension (self-report)**										
No	600	54.7	906	61.4		434	55.9	638	61.7	
Yes	496	45.3	569	38.6	0.001	342	44.1	396	38.3	0.01
**Family History of Cancer** 3										
No	733	66.8	1,074	72.8		512	65.9	745	72.0	
Yes	364	33.2	402	27.2	0.001	265	34.1	290	28.0	0.01
**Vegetable Intake** 4										
Low	362	34.0	420	29.3		251	33.2	288	28.6	
Medium	426	40.0	542	37.8		311	41.1	391	38.8	
High	277	26.0	471	32.9	0.001	194	25.7	329	32.6	0.005
**Alcohol intake** 5										
Non-drinkers	283	25.8	349	23.6		201	25.9	243	23.5	
Low	318	29.0	376	25.5		218	28.1	297	28.7	
Medium	296	27.0	370	25.1		199	25.6	248	24.0	
High	198	18.1	381	25.8	<0.001	158	20.4	247	23.9	0.25

1 Chi-square test for proportions; T-test for means.

2 Four centers: Brno, Olomouc, Prague, Ceske-Budejovice.

3 Cancer in first-degree relatives.

4 Tertiles based on frequency of vegetable intake among controls.

5 Tertiles for drinkers, based on weighted average of consumption.

Results from the individual SNP and gene-based minimum p-value permutation (min-P) tests are shown in Table 1. The strongest associations with RCC risk were observed for *SLC19A1* (P_min-P_ = 0.03) and *MTHFR* (P_min-P_ = 0.13). Two individual SNPs tagging the *SLC19A1* region, rs12483553 and rs17004785, were significantly associated with RCC after adjustment for multiple testing (FDR-adjusted P_trend_ = 0.03 for each). The association for each SNP was slightly attenuated when both were included together in the logistic regression model. The haplowalk analysis also identified *SLC19A1* as a region of interest, with an FDR-adjusted p-value of 0.02 for the most significant 3-SNP window. Based on the haplowalk analysis and the LD structure among control subjects, we identified a block of four tag SNPs to examine in haplotype analysis (rs12483553, rs2838950, rs2838951, and rs17004785). The A-C-C-C haplotype was associated with a significant 37% increased RCC risk compared to the most frequent haplotype, G-C-G-G (Table 3). The two SNPs identified in the single SNP analysis appear to be driving the haplotype result, as other haplotypes that did not include the minor allele for rs12483553 or rs17004785 did not have different RCC risks relative to the reference haplotype. After stratification, we observed that both the individual SNPs and the A-C-C-C haplotype were significantly associated with RCC risk only among those in the lowest tertile of vegetable intake (Table 4). The overall distribution of *SLC19A1* haplotypes differed significantly (P_global_ = 0.05) between cases and controls within the lowest tertile of vegetable intake, but not within the middle or highest tertile. Power was limited, and tests for interaction between individual haplotypes and vegetable intake did not yield any significant interactions (Table 4). No differences in haplotype distributions or individual SNP effects were observed in analyses stratified by alcohol intake for any of the genes examined.

**Table 3:** Haplotypes in selected one-carbon metabolism genes and risk of renal cell carcinoma.

Haplotypes	Cases (%)	Controls (%)	OR^1^	(95% CI)	P-value	Global P-value
**SLC19A1**						
Region 1						
5′ - rs12483553, rs2838950, rs2838951, rs17004785 - 3′						
**G-C-G-G**	42.5	43.8	1.00	(ref)		
**A-C-C-C**	8.4	6.4	1.37	(1.05–1.78)	0.02	
**G-C-C-G**	20.6	21.0	1.00	(0.83–1.20)	0.99	
**G-T-C-G**	22.8	24.2	0.97	(0.83–1.15)	0.74	
**Rare**			1.20	(0.87–1.64)	0.27	
						0.13
**MTHFR**						
Region 2						
5′ - rs12121543, rs1801133, rs17421511, rs11121832, rs9651118 - 3′						
**G-T-C-G-A**	33.9	30.2	1.00	(ref)		
**T-C-T-A-A**	18.0	17.6	0.92	(0.75–1.12)	0.40	
**T-C-C-G-A**	10.2	10.1	0.91	(0.72–1.16)	0.44	
**G-C-C-G-G**	23.2	24.4	0.86	(0.72–1.02)	0.09	
**G-C-C-G-A**	5.5	7.0	0.69	(0.51–0.93)	0.01	
**G-C-C-A-A**	7.3	9.4	0.69	(0.53–0.90)	0.01	
**Rare**			1.31	(0.71–2.40)	0.39	
						0.04

1 Adjusted for age, sex, and study center.

**Table 4:** Haplotypes in selected one-carbon metabolism genes and risk of renal cell carcinoma, stratified by frequency of vegetable intake.

	Tertiles of vegetable intake frequency						
	Low		Medium		High		
Haplotypes	OR^1^	95% CI	OR^1^	95% CI	OR^1^	95% CI	P-int^2^
**SLC19A1**							
Region 1							
5′ - rs12483553, rs2838950, rs2838951, rs17004785 - 3′							
**G-C-G-G**	1.00	(ref)	1.00	(ref)	1.00	(ref)	
**A-C-C-C**	1.92	(1.17–3.15)	1.05	(0.70–1.58)	1.31	(0.75–2.27)	0.19
**G-C-C-G**	0.96	(0.69–1.34)	0.95	(0.71–1.28)	0.98	(0.69–1.38)	0.85
**G-T-C-G**	0.91	(0.67–1.23)	0.91	(0.69–1.18)	1.03	(0.76–1.40)	0.67
**P-global**	0.05		0.91		0.74		
**rs12483553** 3	1.81	(1.19–2.76)	1.11	(0.77–1.58)	1.32	(0.83–2.11)	0.26
**rs17004785** 3	2.05	(1.36–3.11)	1.06	(0.76–1.48)	1.30	(0.85–2.01)	0.09
**MTHFR**							
Region 2							
5′ - rs12121543, rs1801133, rs17421511, rs11121832, rs9651118 - 3′							
**G-T-C-G-A**	1.00	(ref)	1.00	(ref)	1.00	(ref)	
**T-C-T-A-A**	0.78	(0.54–1.15)	1.10	(0.80–1.51)	0.89	(0.61–1.29)	0.63
**T-C-C-G-A**	0.78	(0.49–1.23)	1.10	(0.76–1.59)	0.89	(0.56–1.41)	0.53
**G-C-C-G-G**	0.73	(0.53–1.01)	0.93	(0.70–1.23)	0.96	(0.68–1.35)	0.21
**G-C-C-G-A**	0.72	(0.41–1.25)	0.61	(0.38–0.97)	0.87	(0.49–1.55)	0.72
**G-C-C-A-A**	0.55	(0.33–0.91)	0.73	(0.48–1.12)	0.79	(0.47–1.30)	0.32
**P-global**	0.34		0.20		0.89		

1 Adjusted for age, sex, and study center.

2 Interactions tested using a likelihood ratio test and adjusting for age, sex, and center.

3 Single SNP analysis.

We utilized data from a recent genome-wide association study (GWAS) of renal cancer [22] with over 3700 Caucasian cases and 8400 controls to look specifically at the association between the two significant *SLC19A1* SNPs identified in our study and RCC risk in the GWAS population. Both SNPs were associated with RCC risk (p = 0.01 for rs12483553; p = 0.04 for rs17004785), though not at a level sufficient for genome-wide significance in an agnostic GWAS examination.

A previous analysis by Moore et al. in the CEERCC study population found a significant association between the known functional SNP in *MTHFR* (rs1801133, 677C>T) and RCC risk [5]. We expanded on the previous result by comprehensively examining the gene region to evaluate whether additional variants in the gene were associated with RCC risk, but none were significant in the single SNP analysis. We identified a region of interest based on the haplowalk analysis and tested haplotypes to examine whether they provided additional information beyond the 677C>T SNP (Table 3). Two haplotypes were found to be significantly associated with RCC risk, but the results are consistent with variation at 677C>T driving the association. Stratification by vegetable intake showed that two *MTHFR* haplotypes were associated with RCC risk in the low or middle tertiles (Table 4), while no haplotypes were significant among those in the highest tertile. However, the effect of vegetable intake on genotype was relatively weak, as the global p-values for the haplotypes were not significant in any vegetable tertile and the tests of interactions had low power but were not significant (Table 4).

No significant associations were observed for individual SNPs or the gene-based min-P test for the other one-carbon genes genotyped in this study (Table 1). Therefore we did not examine specific haplotypes for these genes. Results for the individual analyses of all SNPs can be found in Table S1.

## Discussion

We conducted an analysis of 163 tag SNPs in 13 genes related to one-carbon metabolism, folate transport, and glutathione synthesis in relation to risk of renal cell carcinoma. We identified one novel gene region that was significantly associated with RCC risk. Specifically, a region proximal to the coding region of *SLC19A1* was found to contain two SNPs that were associated with risk of RCC, and a haplotype containing the minor allele for both these SNPs was associated with a significant 39% increased risk. The association was particularly evident among participants who reported low vegetable intake, which may be a proxy for folate intake/status. Supporting our findings, the two significant *SLC19A1* SNPs in our study were also associated with RCC in a recent GWAS study of renal cancer [22], though not at a level of genome-wide significance. Our results are novel and require replication in other studies that can examine vegetable or folate intake.

There is a biologic rationale for an involvement of the *SLC19A1* gene in cancer risk. *SLC19A1* codes for the reduced folate carrier (RFC), which transports reduced folates and antifolate drugs into mammalian cells [23]. Humans are unable to synthesize folates, which are at the foundation of the reactions of one-carbon metabolism, so adequate folate must be consumed, effectively absorbed, and transported to cells and tissues. RFC is ubiquitously expressed, including at high levels in the kidney, and is the major transport system for folates in most tissues [23]. In addition to uptake of folate from the blood, RFC is also involved in absorption of folate in the intestine as well as transport across the basolateral membrane of the renal tubules [23]. Genetic variations in *SLC19A1* could result in differing RFC activity and therefore differing folate availability. Folate deficiency has been associated with a number of cancers, including RCC, through mechanisms thought to involve its role in the provision of substrates for DNA synthesis and methylation [2]–[5].

Existing evidence for an association between *SLC19A1* and cancer risk is sparse. Mouse models suggest that levels of RFC can dramatically impact the neoplastic processes leading to colorectal cancer [23]. Variation in *SLC19A1* has not been studied in relation to renal cancer, but epidemiologic studies have examined the association of this gene with other cancers. Most studies examined one particular nonsynonymous SNP (rs1051266, 80G>A), which has been associated with changes in blood concentrations of folate in some studies [24], [25] but not others [26], [27]. We did not have genotype information for this SNP, though it was moderately correlated with one of our tag SNPs (rs2838951; r^2^ = 0.59). Other studies have found possible associations for rs1051266 with risk of bladder, esophageal, and lung cancers [28]–[30], but not with colon, prostate, and breast cancers [31]–[34]. A recent study examined tag SNPs of one-carbon genes and found five SNPs in *SLC19A1* that were significantly associated with risk of colorectal adenomas, including rs1051266 [35]. One of the specific *SLC19A1* tag SNPs found to be associated with RCC risk in our study, rs17004785, was not associated with risk in the studies of colorectal cancer and colorectal adenomas [31], [35].

Haplotypes in both *SLC19A1* and *MTHFR* were found to be significantly associated with RCC risk among participants in the lowest tertile of vegetable intake frequency, but no effect of genotype was observed among those in the highest tertile. Vegetables are good sources of folate, particularly in populations such as Eastern and Central Europe where grain products are not fortified with folic acid. Thus vegetable intake may be a rough proxy for folate intake, in which case our results suggest that the effect of genotype for these folate-associated genes may be important only when folate intake is low. A number of studies of *MTHFR* and cancer risk have demonstrated an increased role of genetic variation under low-folate conditions [10], [36], and numerous examples support the theory that diet can modify the impact of genetic variation [37].

To our knowledge, this is the first study to comprehensively examine variation in one-carbon metabolism and folate transport genes in relation to renal cancer risk, including examination of different dietary intakes of folate sources. We used HapMap data with a tag SNP approach to achieve high (80–90%) genomic coverage of the genes of interest, and examined regions upstream and downstream of the coding regions. In addition, the study was conducted in a population without mandatory fortification of grain products with folic acid; similar studies in fortified populations with uniformly high folate intake may not be able to fully examine the interaction between folate intake and genetic effects related to folate transport and metabolism. Other strengths of our study were inclusion of only newly diagnosed and histologically confirmed renal cancers, a high participation rate, and collection of biological samples from a high proportion of participants. The multi-stage analysis and correction for multiple testing reduced the possibility of Type I error. Population stratification is a possible concern, but we found no evidence of heterogeneity by country, and bias from population stratification is unlikely to be substantial in studies of cancer in non-Hispanic European populations [38]. A limitation of our study is that the dietary assessment instrument in the CEERC study was designed specifically to assess intake of the foods most frequently consumed in the study region, but it did not capture the quantity of foods consumed. Diet was assessed after case diagnosis, so recall bias is possible if case status influenced how participants recalled their dietary habits. Given the limitations in the assessment of diet and the need for replication, our analyses should be considered exploratory.

In conclusion, we comprehensively examined genetic variation in 13 genes associated with one-carbon metabolism using a tag SNP approach and identified a novel association between *SLC19A1* and renal cell cancer. Additional studies are needed to replicate these findings and better understand the impact of folate intake.

## Supporting Information

Figure S1 **Reactions of one-carbon metabolism.** A diagram showing the reactions of one-carbon metabolism (simplified), including the role of gene products from the 12 candidate genes in the analysis (*GGH* not shown) and associated vitamin cofactors. Numbers in brackets [#] correspond to genes listed in Table 1.
(DOC)

Table S1 **Main effects SNP-based analyses for genes in the one-carbon metabolism pathway and RCC risk.**
(XLS)
