# Supplementary material for: Comprehensive Evaluation of One-Carbon Metabolism Pathway Gene Variants and Renal Cell Cancer Risk
Source: PLoS One. 2011 Oct 19;6(10):e26165. doi: 10.1371/journal.pone.0026165 (PMC3198392; doi:10.1371/journal.pone.0026165)
Supplement: Figure S1 — Reactions of one-carbon metabolism. A diagram showing the reactions of one-carbon metabolism (simplified), including the role of gene products from the 12 candidate genes in the analysis (GGH not shown) and associated vitamin cofactors. Numbers in brackets [#] correspond to genes listed in Table 1. (DOC) [file pone.0026165.s001.doc]

**[1]**

**[2]**

**[3]**

**[4]**

**[5]**

**[6]**

**[7]**

**[8]**

**[9]**

**[10]**

**[11]**

**[12]**

**[NS]** GGH not shown in figure

**[8]**

**[2]**
